# The Contribution of Genetic Risk and Lifestyle Factors in the Progression of Diabetes to Diabetic Kidney Disease: A Prospective Cohort Study

**DOI:** 10.1111/1753-0407.70141

**Published:** 2025-09-10

**Authors:** Yujiao Wang, Chunyang Li, Nongbu Cili, Jing Chen, Huazhen Yang, Ping Fu, Xiaoxi Zeng

**Affiliations:** ^1^ Division of Nephrology, Kidney Research Institute West China Hospital of Sichuan University Chengdu Sichuan China; ^2^ West China Biomedical Big Data Center West China Hospital of Sichuan University Chengdu Sichuan China; ^3^ Department of Nephrology Affiliated Hospital of Zunyi Medical University Zunyi Guizhou China

**Keywords:** diabetes, diabetic kidney disease, genetic risk, lifestyle

## Abstract

**Aims:**

Diabetes is a global public health crisis, especially when it is accompanied by microvascular complications such as diabetic kidney disease (DKD). The purpose of this study was to explore the relationship between the combined lifestyle factors of diabetes patients and their joint effects with genetic risk and the risk of DKD.

**Materials and Methods:**

We included individuals diagnosed with diabetes at baseline from UK Biobank. Their lifestyle information was collected through a baseline questionnaire. Favorable lifestyle scores were constructed based on 5 common lifestyle factors and categorized into three levels. Genetic susceptibility to CKD was estimated by polygenic risk scores and further categorized into high, and low genetic risk categories. Cox proportional hazards regression model was used to estimate the hazard ratios (HR) and 95% confidence interval (CI) for their associations.

**Results:**

By the end of follow‐up, 1335 of 11 981 diabetes patients progressed to diabetes nephropathy. The COX regression results indicate that BMI ≥ 25 mg/m^2^ and current or past smoking were risk factors for DKD, while alcohol consumption and moderate to high‐intensity pysical exercise were protective factors. High genetic risk is significantly associated with increased risk of DKD (HR1.29, 95% CI 1.13–1.47, *p* < 0.001), while a favorable lifestyle had a protective effect (HR0.47, 95% CI 0.37–0.59, *p* < 0.001). Interaction analysis shows that there was neither additive nor multiplicative interaction between genetic risk and lifestyle.

**Conclusions:**

Lifestyle factors and genetics are independently associated with susceptibility to incident DKD. A healthy lifestyle may attenuate elevated DKD risks due to genetic vulnerability.

## Graphical abstract

The Contribution of Genetic Risk and Lifestyle Factors in the Progression of Diabetes to Diabetes Nephropathy.

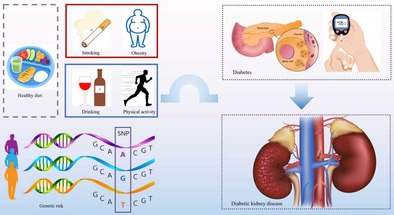

**Summary**

- Through a prospective study based on the UK Biobank, we found that lifestyle factors and genetic factors are independently associated with the risk of developing diabetic kidney disease (DKD).
- A healthy lifestyle may mitigate the elevated risk of DKD due to genetic susceptibility.

## 1 Introduction

Diabetes mellitus (DM) affects more than 500 million adults worldwide, and it is predicted that by 2045, the absolute number of diabetes patients will increase by 46% [1]. Microvascular complications in diabetes are the main cause of poor prognosis, including diabetic retinopathy, diabetic neuropathy, and diabetic kidney disease (DKD) [2, 3]. Among them, DKD occurs in up to 40% of individuals with diabetes and remains an important cause of end‐stage renal disease worldwide, accounting for over 50% of individuals receiving dialysis and kidney transplantation therapy, which has brought enormous health and economic burdens to patients, families, society, and medical institutions [4, 5]. Therefore, it is paramount to identify cost‐effective strategies to prevent and delay the development of DKD in patients with diabetes.

It is worth emphasizing that numerous genetic studies have demonstrated a clear genetic component to both diabetes and its complications [6]. Beyond the glucose control by oral drugs and insulin, the American Diabetes Association guideline has highlighted that how to optimize lifestyle behavior to improve diabetes care is worthy of attention, especially for high‐genetic‐risk groups [7]. Although studies have shown that a healthy diet, non‐smoking, and proper physical activity are associated with a lower risk of microvascular complications, as far as we know, the role of multiple lifestyle factors and their combined association with genetic risk in the occurrence and development of diabetes nephropathy has not been quantitatively studied, which may have substantial public health implications on translating epidemiological findings to meaningful public health actions [8]. Therefore, based on the UK Biobank database, we investigated the joint association of multiple lifestyle behaviors, including body mass index (BMI), smoking status, alcohol consumption status, dietary habits, and physical exercise with the risks of DKD in individuals with diabetes. Besides, after confirming the association between the polygenic risk score(PRS) for estimated glomerular filtration rate (eGFR) and the development of DKD in diabetes, we analyzed the combined effects of different genetic risks and lifestyles, aimed to guide patients to adjust their lifestyle and develop healthy prevention strategies to reduce the disease burden of DKD.

## 2 Materials and Methods

### 2.1 Study Population

This cohort study is based on data collected from the UK Biobank, which is a national prospective cohort with very large and detailed data from over 500 000 participants aged 40 to 69 years when recruited at baseline (in 2006–2010) across England, Wales, and Scotland, which ensured a wide distribution across all exposures to provide reliable associations between personal characteristics and health outcomes [9].

**FIGURE 1:**
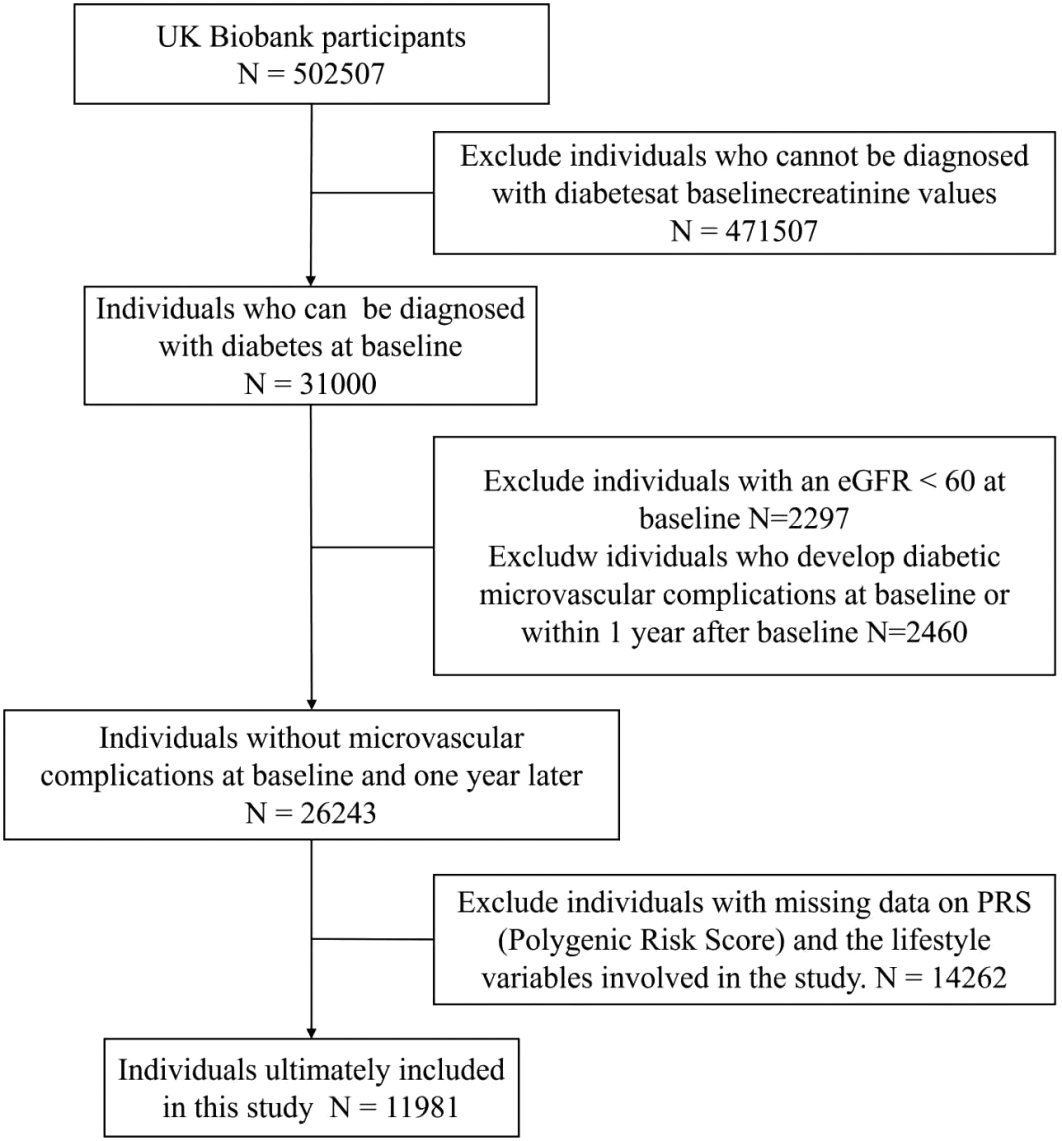
Flow chart of participants' enrollment.

Among 502 507 participants from the UK Biobank, we included 31 000 individuals who were diagnosed with diabetes at the baseline survey. The diagnosis could be made if any of the following criteria were met: (1) a previous inpatient diagnosis of diabetes; (2) self‐report diagnosis of diabetes; (3) baseline glycated hemoglobin (HbA1c) ≥ 6.5%; (4) baseline random blood glucose ≥ 11.1 mmol/L. Subsequently, we excluded individuals with eGFR < 60 mL/min/1.73m^2^ at baseline, or those who developed diabetic microvascular complications before baseline or within the first year after baseline. The codes for the definitions of diabetes and related complications were listed in Table S1. We further excluded individuals who lacked the qualified genetic data and lifestyle data required for our study. Ultimately, a total of 11 981 individuals were included in this study (Figure 1).

### 2.2 Cases Ascertainment and Follow‐Up

Incident cases with DKD were ascertained by International Classification of Diseases version 10 (ICD‐10) diagnoses in hospital inpatient records and death registry (Table S1). Participants were followed up from the baseline until the first diagnosis of DKD, the date of death, and end of follow‐up, whichever came first.

### 2.3 Genetic Risk Profiling

Genotyping in the UK Biobank was performed using the UK BiLEVE AxiomTM Array and UK Biobank Axiom array from Affymetrix, and variants were imputed using Haplotype Reference Consortium, as well as merged UK10K and 1000 Genomes phase 3 reference panels [10]. Herein, GWAS summary statistics of eGFR based on serum creatinine of CKD Genetics (CKDGen) consortium were used as base data for PRS calculation. Calculation of individual PRS were performed by using PLINK1.9 software (For specific details, please refer to our previous research) [11]. Finally, we used PRS with good stratification ability to represent the genetic susceptibility of CKD, and further used it to classify low (lowest quartile), moderate (second quartile), and high (highest quartile) genetic risk groups.

### 2.4 Modifiable Lifestyle Factors and Covariates

We explored the relationship between five variable lifestyle factors and the risk of diabetes progressing to DKD, including BMI (classified as ≥ 25 kg/m^2^ for the relative obese group verse < 25 kg/m^2^ for the normal group), diet (healthy versus unhealthy), smoking status (never smoked versus other, including current and former smokers), alcohol consumption (never drank versus other, including moderate and excessive drinkers), and physical exercise levels (low versus other, encompassing moderate and high levels). Covariates included age, sex, educational level (A_O_CSE, NVQ_HND_HNC_professiol, college, missing values), income (low, medium, high, missing values), employment status (unemployment, employment), Townsend deprivation index (TDI), comorbidity with hypertension (yes, no), DM duration (< 3 years, ≥ 3 years) and HbA1c level (< 7%, ≥ 7%). Among them, the last two variables primarily reflect the severity of diabetes. Detailed definitions for these lifestyle factors and covariates were shown in the Table S2. The favorable lifestyle score is constructed based on the aforementioned lifestyle factors. Through preliminary analysis, we can determine which lifestyle is relatively beneficial for diabetics. Those with a favorable lifestyle will receive 1 point. The higher the lifestyle score, the higher the level of adherence to a healthy lifestyle. Finally, based on the scores, they were divided into three groups: favorable (4 or 5 healthy lifestyle factors), intermediate (3 healthy lifestyle factors), and unfavorable (0–2 healthy lifestyle factors) lifestyle groups.

### 2.5 Statistical Analysis

Baseline characteristics are presented in groups according to whether they progress to diabetic nephropathy. Continuous variables were shown as means with standard deviation (SDs) if normally distributed and medians with interquartile ranges (IQR) if skewed. Categorical variables were displayed as count with percentage (%). Between‐group differences in baseline characteristics were compared for continuous variables using analysis of variance or Kruskal–Wallis test, as appropriate, and for categorical variables using chi‐squared test. We used a Cox proportional hazards model to analyze the associations between lifestyle/genetic risk category and incidence of DKD, with follow‐up time as the time scale. Model 1 presents the results of a univariate analysis without adjustment for covariates. Model 2 adjusts for age (continuous) and sex; Model 3 further adjusts for educational level, income, employment status, TDI, and Model 4 corrects for comorbidity with hypertension, DM duration, and HbA1c level on the basis of Model 3. A *p* value for trend was calculated treating categorical variables as a continuous variable. The cumulative incidence of DKD by categories of genetic risk and lifestyle categories was obtained using the cumulative incidence function of competing risk regression [12]. Given the differences in lifestyles between genders, we conducted subgroup analyses stratified by sex. In addition, considering that another commonly used threshold for obesity is BMI of 30 kg/m^2^, we re‐categorized individuals into different groups based on this BMI cutoff of 30 for the purpose of conducting a sensitivity analysis. Given that the spot urine albumin‐to‐creatinine ratio (UACR) was assessed only once at baseline, and considering that the false‐positive rate of UACR increases with age and that UACR may not be suitable as a definitive diagnostic test [13], we did not include UACR as a criterion for excluding prevalent CKD at baseline. Instead, in sensitivity analyses, we excluded 498 individuals who either had missing UACR values or a UACR ≥ 30 mg/g at baseline to confirm the robustness of our findings.

By employing the Mover method to calculate the attributable proportion (AP) due to additive interaction and testing whether the relative excess risk due to interaction (RERI) is equal to zero, we analyzed the additive interplay between genetic risk and lifestyle factors [14, 15]. The interactions between lifestyle factors and genetic risk were also examined using a multiplicative interaction model. The cumulative incidence of DKD by categories of genetic risk and lifestyle scores was obtained using the cumulative incidence function of competing risk regression. All tests were 2‐sided, and the association with the *p* value < 0.05 was deemed significant. All analyses were performed using R software, version 4.0.4.

## 3 Result

Of the 11 981 participants, mean age was 59.88, and 35.56% were women. By the end of follow‐up on November 30, 2022, a total of 1335 individuals of 11 981 diabetes patients developed DKD. As shown in Table 1, compared to those who did not develop DKD, patients with DKD were older, had a longer duration of diabetes and higher HbA1c levels, and were more likely to be male, belong to the low‐income group, and had a higher likelihood of comorbid hypertension, while being less likely to have a college degree. The baseline characteristics displayed according to different genetic risk categories and lifestyle categories can be found in Tables S3 and S4.

**TABLE 1:** Baseline characteristics of participants.

Characteristic	No DKD	DKD	*p*
	(*n* = 10 646)	(*n* = 1335)	
Sex (%)			
Female	3786 (35.56)	408 (30.56)	0.0003
Male	6860 (64.44)	927 (69.44)	
Baseline age (median [IQR])	61.02 [54.98, 65.15]	63.60 [59.88, 66.72]	< 0.0001
Age group			
< 45	475 (4.46)	13 (0.97)	< 0.0001
45–60	4236 (39.79)	332 (24.87)	
> 60	5935 (55.75)	990 (74.16)	
TDI (median [IQR])	−1.93 [−3.50, 1.01]	−1.70[−3.38, 1.53]	0.0036
Educational level			
A_O_CSE	3905 (36.58)	434 (32.51)	< 0.0001
NVQ_HND_HNC_profession	1553 (14.59)	227 (17.00)	
College	2849 (26.76)	246 (18.43)	
NA	2339 (21.97)	428 (32.06)	
Income (%)			
Low	2710 (25.46)	498 (37.30)	< 0.0001
Medium	4890 (45.93)	524 (39.25)	
High	1454 (13.66)	108 (8.09)	
NA	1284 (12.06)	190 (14.23)	
Employment status			
Unemployment	1210 (11.37)	157 (11.76)	0.3689
Employment	9436 (88.63)	1178 (88.24)	
Hypertension (%)			
Yes	6724 (63.16)	1059 (79.33)	< 0.0001
No	3922 (36.84)	276 (20.67)	
HbA1c (%)			
< 7%	6344 (62.07)	711 (55.37)	< 0.0001
≥ 7%	3876 (37.93)	573 (44.63)	
DM duration (%)			
< 3	5785 (54.34)	642 (48.09)	< 0.0001
≥ 3 years	4861 (45.66)	693 (51.91)	
BMI type (%)			
25	1309 (12.30)	103 (7.72)	< 0.0001
≥ 25	9337 (87.70)	1232 (92.28)	
Smoking status (%)			
Never	4544 (42.68)	593 (44.42)	0.2383
Other (current or previous)	6102 (57.32)	742 (55.58)	
Drinking status (%)			
Never	1135 (10.66)	131 (9.81)	0.3663
Other(some and too much)	9511 (89.34)	1204 (90.19)	
Diet status			
Healthy	1639 (15.40)	203 (15.21)	0.8881
Unhealthy	9007 (84.60)	1132 (84.79)	
Physical activity level (%)			
Low	2810 (26.39)	334 (25.02)	0.2963
Middle and high	7836 (73.61)	1001 (74.98)	
Number of favorable lifestyle factors			
0	134 (1.26)	14 (1.05)	0.2633
1	1670 (15.69)	211 (15.81)	
2	4501 (42.28)	528 (39.55)	
3	3362 (31.58)	440 (32.96)	
4	877 (8.24)	130 (9.74)	
5	102 (0.96)	12 (0.90)	
Lifestyle category			
Unfavorable	6305 (59.22)	753 (56.40)	0.0844
Intermediate	3362 (31.58)	440 (32.96)	
Favorable	979 (9.20)	142 (10.64)	
Genetic risk category			
Low	3610 (33.91)	383 (28.69)	< 0.001
Moderate	3536 (33.21)	458 (34.31)	
High	3500 (32.88)	494 (37.00)	

**FIGURE 2:**
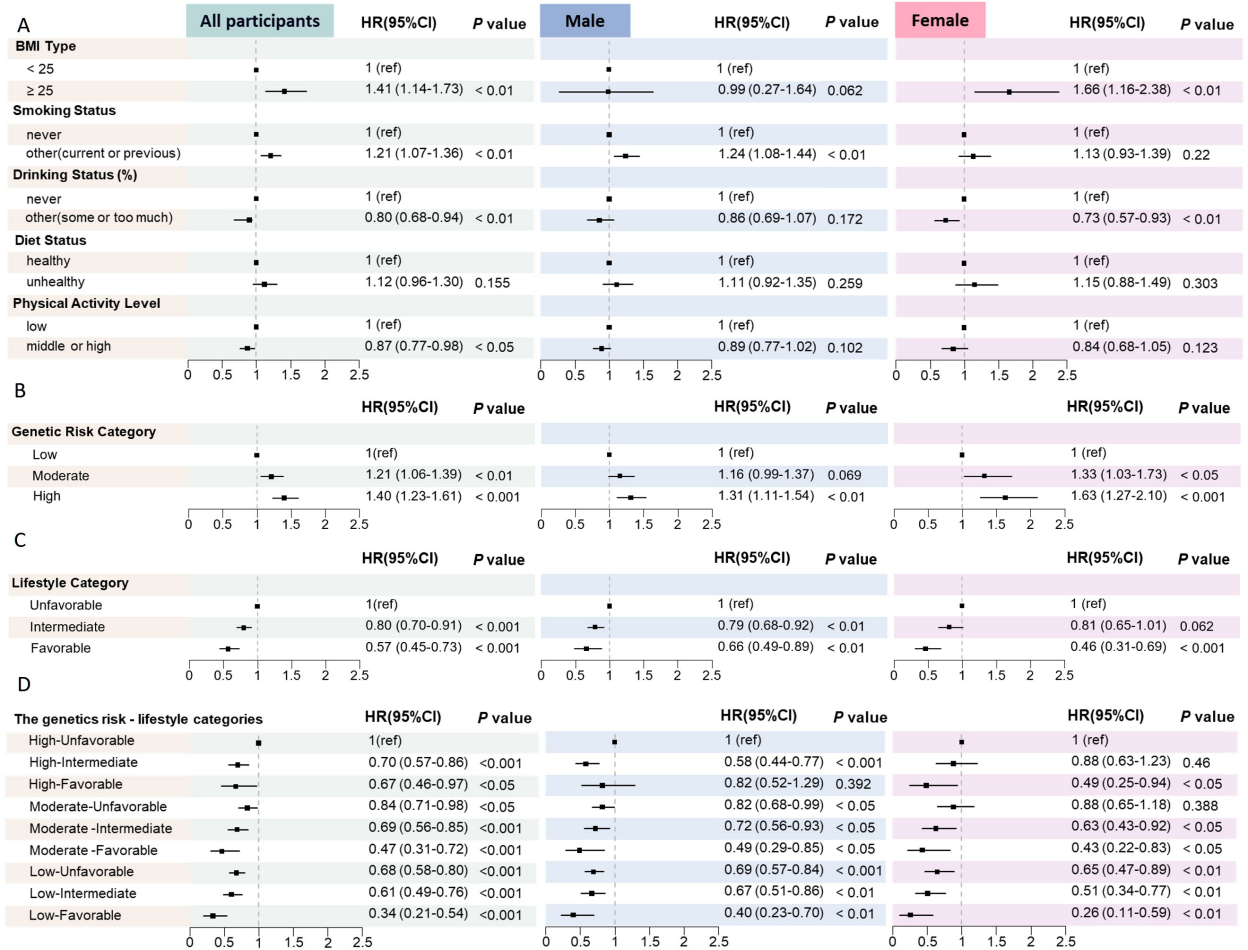
Multifactor regression of DKD risk at different exposure levels for all participants, males, and females, respectively.

We first evaluated the association of genetic risk and individual lifestyle factors with the risk of developing DKD. We found that, compared to individuals with low genetic risk for CKD, individuals with moderate (HR: 1.21, 95% CI 1.06–1.39, *p* < 0.01) and high genetic risk (HR: 1.40, 95% CI 1.23–1.61, *p* < 0.001) were associated with significant risk for CKD in patients with diabetes, after adjusting for age, sex, TDI, income, educational level, employment status, comorbidity with hypertension, DM duration, HbA1c, and level (Table 2, Figure 2). Besides, for individual analysis of each component of lifestyles, BMI ≥ 25 kg/m^2^, current and previous smoking were associated with increased risk with DKD, while regular alcohol consumption and higher physical activity level were associated with decreased risk with DKD (Table 3, Figure 2) (all *p* < 0.05), so we considered drinking alcohol as a favorable lifestyle in the subsequent analysis. But the association with diet status was not statistically significant (*p* = 0.155); nevertheless, given that diet status was a well‐established lifestyle factor related to DKD based on previous evidence [14, 15], we decided to include it for the construction of favorable lifestyle scores.

**TABLE 2:** Risk of DKD according to genetic risk and lifestyle categories.

Category	Events/person‐years	Model 1		Model 2		Model 3		Model 4	
		HR (95% CI)	*p*	HR (95% CI)	*p*	HR(95% CI)	Value	HR (95% CI)	*p*
Genetic risk category									
Low	383/55223.75	1 (ref)		1 (ref)		1 (ref)		1 (ref)	
Moderate	458/55645.81	1.17 (1.02–1.34)	< 0.05	1.20 (1.05–1.37)	< 0.05	1.19 (1.04–1.36)	< 0.05	1.21 (1.06–1.39)	< 0.01
High	494/55320.97	1.29 (1.13–1.47)	< 0.001	1.34 (1.17–1.53)	< 0.001	1.36 (1.19–1.55)	< 0.001	1.40 (1.23–1.61)	< 0.001
*p* value for trend^#^			< 0.001		< 0.001		< 0.001		< 0.001
Lifestyle category									
Unfavorable	900/96479.11	1 (ref)		1 (ref)		1 (ref)		1 (ref)	
Intermediate	360/53386.92	0.71 (0.63–0.80)	< 0.001	0.74 (0.38–0.61)	< 0.001	0.83 (0.74–0.94)	< 0.001	0.80 (0.70–0.91)	< 0.001
Favorable	75/16324.51	0.47 (0.37–0.59)	< 0.001	0.48 (0.66–0.84)	< 0.001	0.63 (0.50–0.81)	< 0.001	0.57 (0.45–0.73)	< 0.001
*p* value for trend^#^			< 0.001		< 0.001		< 0.001		< 0.001

*Note:* Model 1: No adjusted. Model 2: Adjusted for age and sex. Model 3: Adjusted for covariates in model 2 and TDI, income, educational level, employment status. Model 4: Adjusted for covariates in model3 and comorbidity with hypertension, DM duration, HbA1c.

^#^ Encode the ordered categorical variables 1, 2, and 3 as continuous variables and include them in the regression model.

**TABLE 3:** Risk of DKD with each lifestyle factor.

Lifestyle factors	Events/Person‐years	Model 1		Model 2		Model 3		Model 4	
		HR (95% CI)	*p*	HR (95% CI)	*p*	HR (95% CI)	*p*	HR (95% CI)	*p*
BMI type									
< 25	103/20069.19	1 (ref)		1 (ref)		1 (ref)		1 (ref)	
≥ 25	1232/146121.3	1.71 (1.40–2.10)	< 0.001	1.67 (1.38–2.10)	< 0.001	1.60 (1.31–1.96)	< 0.001	1.41 (1.14–1.73)	< 0.01
Smoking status									
Never	473/73083.54	1 (ref)		1 (ref)		1 (ref)		1 (ref)	
Other(current or previous)	862/93016.99	1.47 (1.31–1.64)	< 0.001	1.30 (1.16–1.47)	< 0.001	1.23 (1.10–1.38)	< 0.001	1.21 (1.07–1.36)	< 0.01
Drinking status (%)									
Never	186/17534.36	1 (ref)		1 (ref)		1 (ref)		1 (ref)	
Other(some or too much)	1149/148656.2	0.74 (0.63–0.86)	< 0.001	0.71 (0.61–0.83)	< 0.001	0.79 (0.67–0.93)	< 0.001	0.80 (0.68–0.94)	< 0.01
Diet status									
Healthy	204/26190.32	1 (ref)		1 (ref)		1 (ref)		1 (ref)	
Unhealthy	1131/140000.2	1.06 (0.92–1.23)	0.419	1.13 (0.98–1.32)	0.103	1.13 (0.97–1.32)	=0.104	1.12 (0.96–1.30)	0.155
Physical activity level									
Low	388/43389.7	1 (ref)		1 (ref)		1 (ref)		1 (ref)	
Middle or high	947/122800.8	0.87 (0.77–0.98)	< 0.05	0.83 (0.74–0.94)	< 0.005	0.85 (0.76–0.96)	< 0.01	0.87 (0.77–0.98)	< 0.05

*Note:* Model 1: No adjusted. Model 2: Adjusted for age and sex. Model 3: Adjusted for covariates in model 2 and TDI, income, educational level, employment status. Model 4: Adjusted for covariates in model3 and comorbidity with hypertension, DM duration, HbA1c.

**FIGURE 3:**
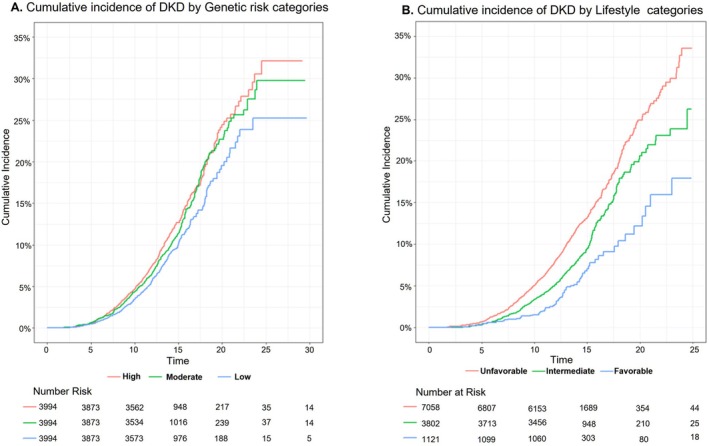
Cumulative incidence of DKD by genetic risk categories and lifestyle categories.

For the combined analysis of lifestyles, when treating as a continuous variable, increasing favorable lifestyle number was associated with lower risks of DKD in a dose–response manner; for each one‐score increment, it was associated with a 22% lower risk (HR: 0.78; 95% CI: 0.73–0.83, *p* < 0.001) (Table S5A). Similarly, as the number of unfavorable lifestyle factors increases, the risk of developing DKD also rises; for each one‐score increment, it was associated with a 29% higher risk (HR: 1.29; 95% CI: 1.21–1.37, *p* < 0.001) (Table S5B). We further assigned participants into unfavorable (number ≤ 2), intermediate (number = 3) and favorable (number ≥ 4) lifestyle categories. The percentage of different combinations of lifestyle factors was listed in Figure S1. Then, we further confirmed that, compared with those who maintained an unfavorable lifestyle, individuals who adhered to a favorable lifestyle exhibited a lower risk of DKD (HR: 0.47, 95% CI 0.37–0.59, *p* < 0.001), and this association remained statistically significant even after model adjustment (HR: 0.57, 95% CI 0.45–0.73, *p* < 0.001) (Table 2, Figure 2). The cumulative incidence rate of DKD during the follow‐up was higher in the group with an unfavorable lifestyle compared with the group with a favorable lifestyle; the same applies to genetic risk groups (Figure 3).

**FIGURE 4:**
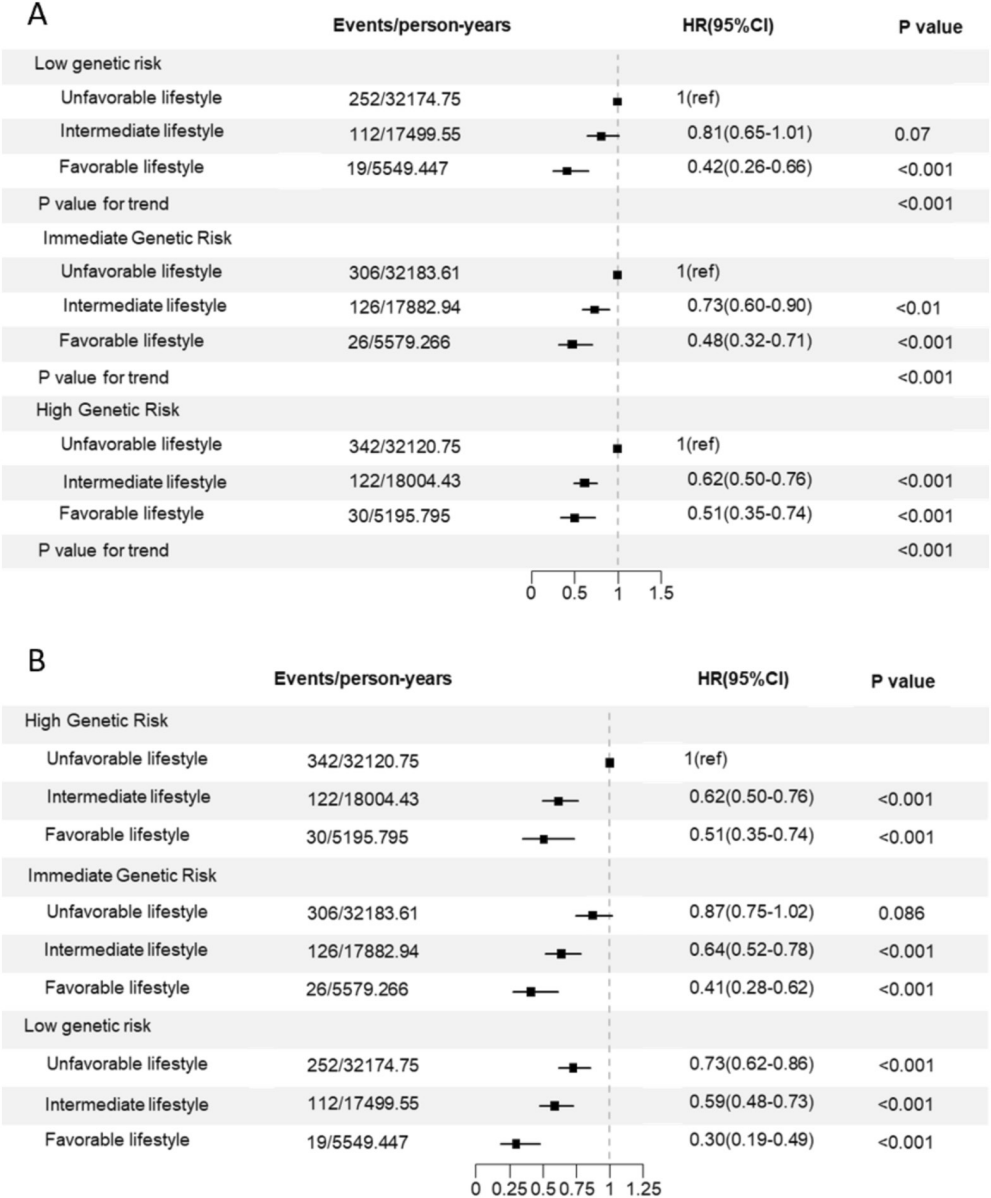
(A) Risk of DKD according to lifestyle categories within each genetic risk category. (B) Risk of DKD by joint categorization for genetic risk and healthy lifestyle.

We then examined the potential interaction between genetic risk and lifestyles on the development of DKD in diabetes. In univariate analysis, for those with low genetic risk, only a favorable lifestyle was related to a lower incidence of DKD (HR: 0.42, 95% CI: 0.26–0.66, *p* < 0.001). For individuals with moderate and high genetic risk, both an intermediate lifestyle and a favorable lifestyle were associated with a decreased risk of developing DKD, compared to an unfavorable lifestyle (Figure 4A). After adjusting for the covariates, a favorable lifestyle was consistently related to a lower incidence of DKD across all genetic risk groups (all *p* < 0.05). However, while an intermediate lifestyle was associated with a reduced risk of DKD in the high genetic risk population, the association was not statistically significant in the moderate genetic risk population (Figure S2A). In the joint analysis of both genetic risk and lifestyles, with individuals of both high genetic risk and the most unhealthy lifestyle as the reference, the lowest estimate of risk was observed for those with both low genetic risk and adopting healthy lifestyles (HR: 0.30, 95% CI: 0.19–0.49, *p* < 0.001). We also found that, despite having a lower genetic risk, if individuals in the low PRS category adopted an unhealthy lifestyle, their risk for DKD remained similar to that of those with a higher genetic risk (HR: 0.73, 95% CI: 0.62–0.86, *p* < 0.001), while for individuals with high genetic risk, following healthy lifestyles was associated with a markedly decreased risk of DKD (Figure 4B). The aforementioned associations remained statistically significant after adjusting for covariates (Figure S2B). We did not detect any multiplicative interaction (*p* = 0.4003) or additive interaction (Relative excess risk due to interaction: 0.08; the attributable proportion due to interaction: 0.09).

The results of the subgroup analysis by gender are presented in Figure 2 and Table S6. Regarding individual lifestyle factors, only the association of smoking with increased risk of DKD remained statistically significant for men after model adjustment (HR: 1.24, 95% CI: 1.08–1.44, *p* < 0.01). For women, a higher BMI was identified as a potential risk factor (HR: 1.66, 95% CI: 1.16–2.38, *p* < 0.01), while alcohol consumption continued to be a protective factor (HR: 0.73, 95% CI: 0.57–0.93, *p* < 0.01). A joint analysis of lifestyle categories and genetic risk revealed that adopting a favorable lifestyle can still reduce the risk of DKD, even among individuals at high genetic risk.

The results of the sensitivity analysis with respect to BMI are shown in Table S7. Compared with a BMI < 30 kg/m^2^, BMI ≥ 30 kg/m^2^ exhibits a significant association with increased risk of DKD. In different genetic subgroups, the protective effect of a healthy lifestyle remains statistically significant. The findings from the joint analysis indicate that adopting a healthy lifestyle can significantly reduce disease risk, even among individuals with a high genetic risk (HR: 0.48, 95% CI: 0.36–0.63, *p* < 0.001). The results of the sensitivity analysis conducted after further excluding study participants based on their UACR are presented in Table S8. High genetic risk, elevated BMI, and smoking remained significant risk factors, whereas alcohol consumption and higher levels of physical activity continued to exhibit protective effects. Across different genetic risk groups, adherence to a favorable lifestyle was associated with a reduced risk of DKD.

## 4 Discussion

In this population‐based cohort study involving 11 981 participants from the UK Biobank, we constructed PRS and favorable lifestyle scores to explore the associations among genetic susceptibility, modifiable lifestyle factors, and the risk of DKD. The results reveal that a high genetic risk, coupled with adherence to an unfavorable lifestyle, is independently linked to an elevated risk of DKD. Even after adjusting for variables such as age, gender, education level, income, comorbidity with hypertension, and severity of DM, the results continue to show statistically significant differences. Specifically, individuals in the high genetic risk and unfavorable lifestyle groups exhibited a significantly higher cumulative incidence of DKD compared to those in the low genetic risk and favorable lifestyle groups, respectively. Notably, for each incremental improvement in favorable lifestyle behaviors, there was a corresponding 12% reduction in the risk of developing DKD. When comparing individuals with low genetic risk and a favorable lifestyle to those with high genetic risk and an unfavorable lifestyle, the former group exhibited a decreased risk of DKD.

The lifetime risk of kidney disease in people with diabetes is estimated to be 10%–30% [16]. Early heritability studies of DKD identified a strong familial clustering of both type 1 diabetes (T1D) and type 2 diabetes (T2D) related DKD. Specifically, individuals with diabetes whose siblings had DKD were found to have approximately 2 to 4 times the risk of developing DKD compared to those with diabetes whose siblings did not have DKD [6], these results implicating genetic predisposition in the cause of DKD. In recent years, researchers have conducted more and more large‐scale GWAS and Mendelian randomization analysis to explore the genetic variation related to diabetes nephropathy, including UMOD [17], COL4A3 [18], COL20A1 [19], TENM2 [20], SLC47A1 [21], and so forth. The heritability analysis of diabetes nephropathy shows that the variance of 34%–59% in T1D nephropathy is explained by common genetic variations, and the estimated heritability of SNP in T2D is 8%–12%, depending on the stage or phenotype definition of DKD [22, 23].

Although the congenital genetic background is of great significance, the acquired lifestyle is more closely related to the occurrence and development of diabetes nephropathy. Although our results show that there is no significant statistical difference between a healthy diet and reducing the risk of diabetes nephropathy, previous studies have shown that high compliance with the Mediterranean diet is associated with lower DKD risk [24], which is a dietary pattern dominated by plant food. Perhaps because plant‐based foods are rich in vitamins C and E, selenium, beta carotene, alpha tocopherols, and polyphenols, they can effectively reduce oxidative damage to lipids and proteins associated with high filtration [25]. A high MUFA/S ratio, omega‐3 fatty acid content in fish, and dietary fiber from legumes can improve hyperlipidemia, endothelial function, and lower blood pressure [26]. Previous studies have explored the relationship between BMI and renal function, but the results have been vastly different. Most studies indicate that obesity is a risk factor for DKD, possibly attributed to its ability to promote insulin resistance, endothelial dysfunction, thrombosis, the inflammatory response, fibrosis, arteriosclerosis, and so on [27, 28, 29]. However, several studies demonstrate that BMI ≥ 25 kg/m^2^ serves as a protective factor for kidney function deterioration in diabetic patients with stage 3 or 4 chronic kidney disease, while a low BMI is associated with an elevated risk of newly onset DKD in the Chinese population [30, 31]. The latest systematic review and meta‐analysis results show that each 1‐kg/m2 increase in BMI was associated with a 3% increased risk of DKD onset in patients with T2D ([RR: 1.03, 95% CI: 1.01–1.04], *I* ^2^ = 70.07%, *p* < 0.0001) [32]. This is consistent with our research findings. In summary, the predictive effect of BMI on T2D patients may be variable based on factors such as race and residual renal function. Future research should further explore the relationship between BMI and renal function. Smoking is a risk factor for the onset and progression of diabetic nephropathy. It not only hinders blood sugar control but also activates advanced glycation end products, resulting in increased urinary protein excretion in diabetic patients. Studies indicate that smokers are at a 4.5‐fold higher risk of developing proteinuria compared to non‐smokers. Smoking not only triggers diabetic nephropathy but also accelerates its progression, ultimately leading to renal failure [33, 34, 35, 36].

Studies have shown that in type 1 diabetes patients who are at risk of or have been diagnosed with DKD, engaging in regular moderate to intense physical exercise is associated with a reduced incidence and progression of kidney disease, as well as a decreased risk of cardiovascular events and mortality [37]. It primarily mitigates kidney damage by enhancing insulin sensitivity, bolstering lipid metabolism, alleviating inflammation and oxidative stress within the kidneys, among other mechanisms [38, 39, 40]. Contrary to our traditional understanding, numerous studies have demonstrated that moderate alcohol consumption serves as a protective factor for kidney disease [41, 42, 43, 44]. It may be because drinking alcohol raises the concentration of high‐density lipoprotein cholesterol, enhances insulin sensitivity, and decreases platelet aggregation rate as well as fibrinolysis [45, 46]. Simultaneously, ethanol enhances the absorption of polyphenols, which possess antioxidant properties [47]. In addition, the anti‐inflammatory effect of alcohol was evidenced by observing an elevation in serum interleukin‐10 levels and a reduction in serum interleukin‐16 levels among individuals who consumed moderate amounts of alcohol [48]. Research indicates that moderate drinking can be beneficial, but this does not imply that everyone can drink without restraint. There is no scientific consensus on the optimal range of alcohol consumption that provides a protective effect on the human body, and it remains unclear whether this protective effect applies to all individuals with diabetes. Similarly, the universality of all the aforementioned results still needs further investigation.

Our research is one of the earliest studies to explore the relationship between genetic susceptibility combined with lifestyle and the progression of diabetes to DKD. The main advantage of this study is the large sample size and longer follow‐up time of the UK Biobank, which gives our research sufficient statistical power to test interactions. However, this study still has a series of limitations. Firstly, our retrospective cohort design cannot completely rule out reverse causal relationships to make causal inferences. Secondly, lifestyle data comes from self‐reported questionnaire surveys and is only evaluated once at baseline, while lifestyle may change during follow‐up and affect association estimates. Thus, well‐designed prospective studies with regular follow‐up of participants' lifestyle are warranted to confirm the lifestyle‐genetic risk‐DKD relationship. Thirdly, our determination of outcomes was solely based on ICD diagnoses, as we did not have access to eGFR data during the follow‐up period, so misclassification of outcomes caused by unrecorded cases in medical records due to reasons such as failure to seek medical attention in a timely manner may weaken the effectiveness estimation. Fourth, although important known confounding factors have been adjusted in the model, there may be residual confounding factors. Finally, our study only included Caucasians, which limits the generalizability of the results, and the correlation needs to be validated in populations of other races.

## 5 Conclusion

Our research based on the UK Biobank database shows that high genetic risk is associated with the progression of diabetes patients to DKD. However, adherence to overall healthy lifestyle behaviors, including abstaining from smoking, BMI management, adopting a healthy diet, engaging in regular physical activity, and moderate alcohol drinking, may substantially mitigate the detrimental effects of genetic vulnerability on incident DKD. Our research results emphasize the importance of public health plans advocating healthy lifestyles and taking a series of relevant interventions in reducing the risk of diabetes nephropathy.

## Author Contributions

Y. W., C. L., N. C. L., and X. Z. conceived and designed the study. C. L. conducted data collection. Y. W. performed the statistical analyses. N. C. L. contributed to the manuscript‐original draft preparation. J. C. and H. Y. critically reviewed and revised the manuscript. P. F. approved the final version to be published. X. Z. was the guarantor of this work and, as such, had full access to all the data in the study and takes responsibility for the integrity of the data and the accuracy of the data analysis.

## Conflicts of Interest

The authors declare no conflicts of interest.

## Supporting information


**Table S1:** The codes for the definitions of diabetes and related complications.
**Table S2:** Definition and explanation of modifiable lifestyle factors and covariates.
**Table S3:** Baseline characteristics of participants according to different genetic risk categories.
**Table S4:** Baseline characteristics of participants according to different lifestyle categories.
**Table S5A:** Risk of DKD with Number of favorable lifestyle factors.
**Table S5B:** Risk of DKD with Number of favorable lifestyle factors.
**Table S6A:** Risk of DKD according to each lifestyle factor, genetic risk and lifestyle categories within the male group.
**Table S6B:** Risk of DKD according to each lifestyle factor, genetic risk and lifestyle categories within the female group.
**Table S7:** The sensitivity analysis with respect to BMI.
**Table S8:** The sensitivity analysis with respect to UACR.
