# Supplementary material for: The Contribution of Genetic Risk and Lifestyle Factors in the Progression of Diabetes to Diabetic Kidney Disease: A Prospective Cohort Study
Source: J Diabetes. 2025 Sep 10;17(9):e70141. doi: 10.1111/1753-0407.70141 (PMC12423428; doi:10.1111/1753-0407.70141)
Supplement: Supplementary file 1 — Table S1: The codes for the definitions of diabetes and related complications. Table S2: Definition and explanation of modifiable lifestyle factors and covariates. Table S3: Baseline characteristics of participants according to different genetic risk categories. Table S4: Baseline characteristics of participants according to different lifestyle categories. Table S5A: Risk of DKD with Number of favorable lifestyle factors. Table S5B: Risk of DKD with Number of favorable lifestyle factors. Table S6A: Risk of DKD according to each lifestyle factor, genetic risk and lifestyle categories within the male group. Table S6B: Risk of DKD according to each lifestyle factor, genetic risk and lifestyle categories within the female group. Table S7: The sensitivity analysis with respect to BMI. Table S8: The sensitivity analysis with respect to UACR. [file JDB-17-e70141-s001.docx]

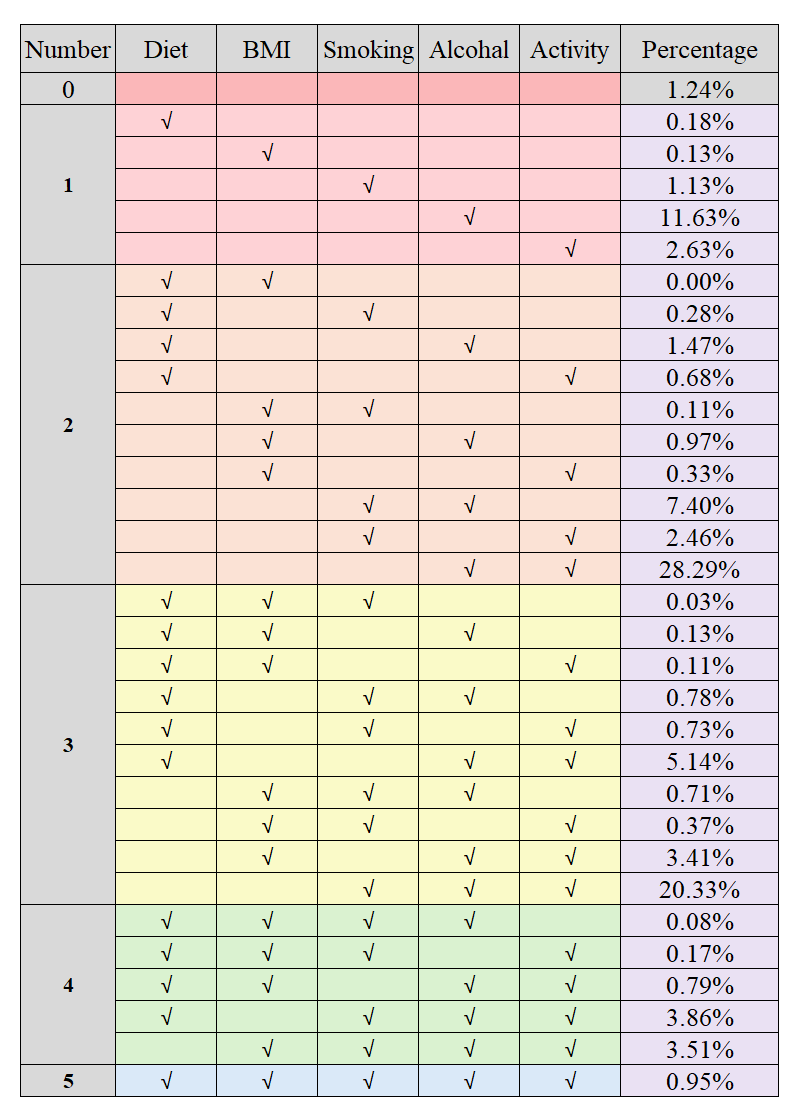


Supplement Figure 1. The constituent ratios of different numbers of favorable lifestyle factors


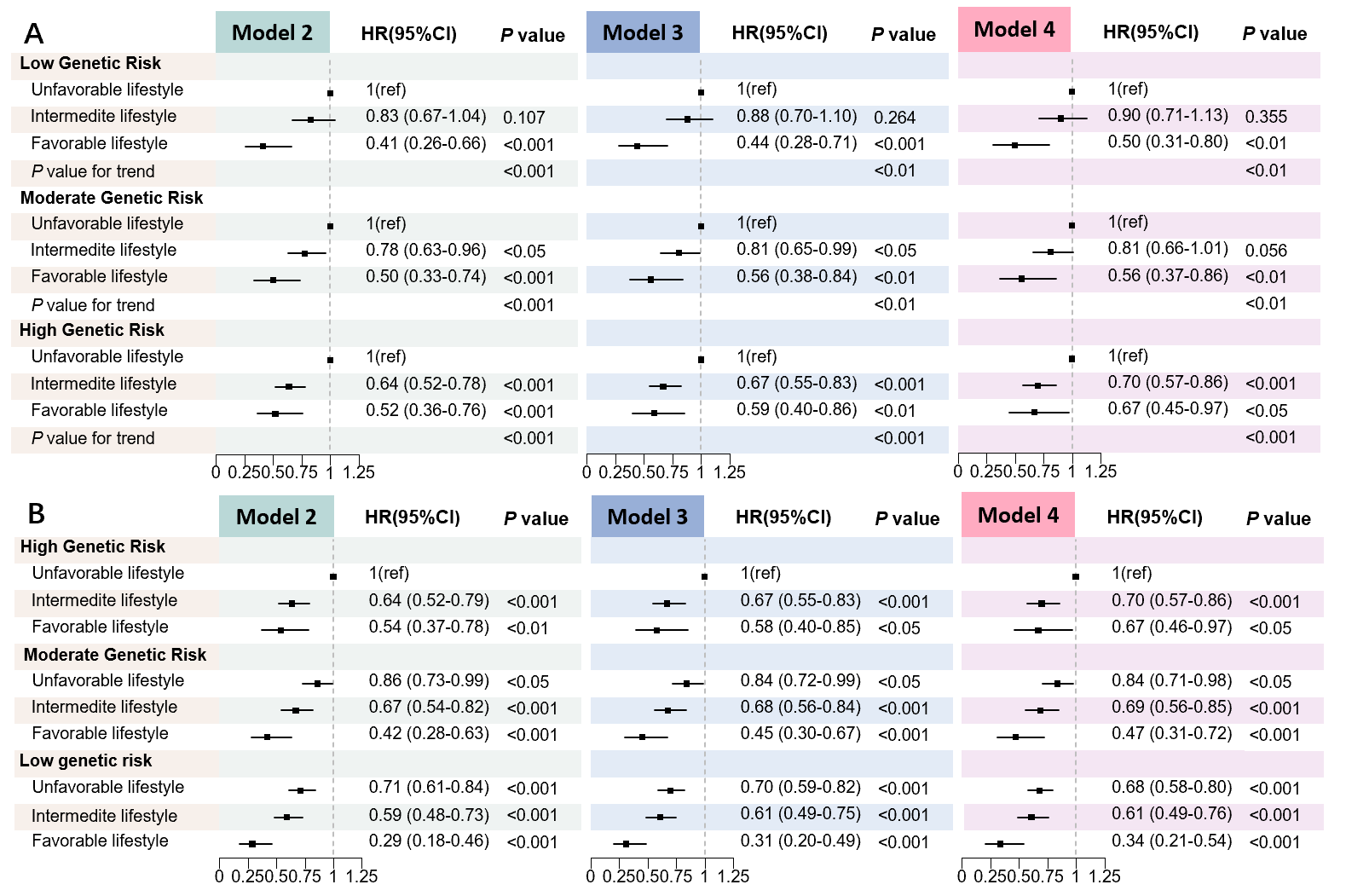


Supplement Figure 2A. Multivariable Cox regression results for the risk of DKD categorized by lifestyle within each genetic risk category.

Supplement Figure 2B. Multivariable Cox regression results for the risk of DKD based on joint categorization of genetic risk and healthy lifestyle.

Supplementary Table1. The codes for the definitions of diabetes and related complications

| Definition | Codes |
| --- | --- |
| Diabetes | ICD-9(250) ICD-10(E10-E14) 2443(1), 20002(1220, 1222, 1223), 6153(3), 6177(3) |
| HbA1c | 30750 |
| Glucose | 30740 |
| Diabetic Kidney Disease | ICD-9(2503) 20002(1607) ID-10 or death cause:E102, E112, E122, E132, E142, N180-185, N188, N189 |
| Diabetic retinopathy | ICD-9(2504) ICD-10:E103, E113, E123, E133, E143, H280, H360 20002(1276) 2443(1), 20002(1220, 1222, 1223), 6153(3), 6177(3) |
| Diabetic neuropathy | ICD-9(2505) ICD-10 or death cause(E104, E114, E124, E134, E144, G590, G629, G632, G990) |

Supplementary Table 2. Definition and Explanation of Modifiable lifestyle factors and Covariates

| Variable | Data fields in the UK Biobank | Descriptions |
| --- | --- | --- |
| Sex | 31 | Classified into women and men |
| Education level | 6138 | Based on the original survey of educational level, it is divided into A_O-CSE or lower college、NVQ_HND_HNC_professiol |
| Household income | 738 | Classified into ultrahigh (>₤100,000), high (₤52,000 to 100,000), medium (₤18,000 to 51,999), and low (< ₤18,000) levels |
| Townsend Deprivation Index | 189 | Treated as a continuous variable. Missing data were imputed by mean values. |
| Body-mass index | 12144, 21002 | Classified into categorized into obesity or overweight(BMI≥25 kg/m2), ,normal or underweight (BMI<25 kg/m2) |
| employment | 6142 | Classified into employed (In paid employment or self-employed, retired, doing unpaid or voluntary work, or full or part-time student) and unemployed (Looking after home and/or family, unable to work because of sickness or disability, or unemployed). |
| Smoking | 20116 | Smoking status was classified into never, previous or current smokers’categories |
| Alcohol consumption |  | Never drinking is defined as "never," "some drinking" is defined as consuming ≤14g of alcohol per day for women and ≤28g for men, and anything above these amounts is considered as "too much." |
| Red wine | 1568, 4407 |  |
| Champagne plus white wine | 1578, 4418 |  |
| Beer plus cider | 1588, 4429 |  |
| Spirits | 1598, 4440 |  |
| Fortified wine | 1608, 4451 |  |
| Other alcoholic | 5364, 4462 |  |
| Diet |  | Considered healthy with at least 5 of the following 10 food groups (Algorithm modified from Said, et al; JAMA Cardiol, 2018, doi: 10.1001/jamacardio.2018.1717) |
| Fruit | 1309, 1319 |  |
| Vegetable | 1289, 1299 |  |
| Whole grains | 1438, 1448 |  |
| Fish | 1329, 1339 |  |
| Dairy | 1408, 1418 |  |
| Vegetable oils | 1428, 2654, 1438 |  |
| Refined grains | 1438, 1448, 1458, 1468 |  |
| Processed meats | 1349, 3680 |  |
| Unprocessed meats | 1359, 1369, 1379, 1389 |  |
| Sugar-sweetened beverage | 6144 |  |
| Physical activity |  | The physical activity was classified into high, middle, and low categories (Algorithm from Said, et al; JAMA Cardiol, 2018, doi: 10.1001/jamacardio.2018.1717) |
| Moderate | 884, 894 |  |
| Vigorous | 884, 894 |  |

Supplementary Table 3. Baseline characteristics of participants according to different genetic risk categories

| Characteristic | Low  (n=3993) | Moderate  (n=3994) | High  (n=3994) | P value |
| --- | --- | --- | --- | --- |
| Sex (%) |  |  |  |  |
| female | 1365 (34.18) | 1393 (34.88) | 1436 (35.96) | 0.2478 |
| male | 2628 (65.82) | 2601 (65.12) | 2557 (64.04) |  |
| Baseline Age (median [IQR]) | 61.60[55.77,65.66] | 61.24[55.51,65.28] | 61.21[55.10, 5.27] | 0.0178 |
| Age Group |  |  |  |  |
| < 45 | 166 (4.16) | 147 (3.68) | 175 (4.38) | 0.2146 |
| 45-60 | 1477 (36.99) | 1550 (38.81) | 1541 (38.58) |  |
| > 60 | 2350 (58.85) | 2297 (57.51) | 2278 (57.04) |  |
| TDI (median [IQR]) | -1.88[-3.50,1.08] | -1.92[-3.50,1.14] | -1.91[-3.49, 1.01] | 0.8293 |
| Educational Level (%) |  |  |  |  |
| A_O_CSE | 1429 (35.79) | 1457 (36.48) | 1453 (36.38) | 0.4198 |
| NVQ_HND_HNC_profession | 617 (15.45) | 576 (14.42) | 587 (14.70) |  |
| college | 1022 (25.59) | 1007 (25.21) | 1066 (26.69) |  |
| NA | 925 (23.17) | 954 (23.89) | 888 (22.23) |  |
| Income (%) |  |  |  |  |
| low | 1104 (27.65) | 1089 (27.27) | 1015 (25.41) | 0.0734 |
| medium | 1752 (43.88) | 1805 (45.19) | 1857 (46.49) |  |
| high | 617 (15.45) | 615 (15.40) | 653 (16.36) |  |
| NA | 520 (13.02) | 485 (12.14) | 469 (11.74) |  |
| Employment status |  |  |  |  |
| unemployment | 431(10.79) | 460 (11.52) | 476 (11.92) | 0.3208 |
| employment | 3562 (89.21) | 3534 (88.48) | 3518 (88.08) |  |
| Hypertension (%) |  |  |  |  |
| yes | 1380 (34.56) | 1365 (34.18) | 1453 (36.38) | 0.088 |
| no | 2613 (65.44) | 2629 (65.82) | 2541 (63.62) |  |
| HbA1c (%) |  |  |  |  |
| < 7% | 2381 (61.59) | 2330 (61.01) | 2344 (61.38) | 0.8709 |
| ≥ 7% | 1485 (38.41) | 1489 (38.99) | 1475 (38.62) |  |
| DM Duration (%) |  |  |  |  |
| < 3 years | 2152 (53.89) | 2139 (53.56) | 2136 (53.48) | 0.9248 |
| ≥ 3 years | 1841 (46.11) | 1855 (46.44) | 1858 (46.52) |  |
| BMI Type (%) |  |  |  |  |
| <25 | 463 (11.60) | 469 (11.74) | 480 (12.02) | 0.8379 |
| ≥25 | 3530 (88.40) | 3525 (88.26) | 3514 (87.98) |  |
| Smoking Status (%) |  |  |  |  |
| never | 412 (10.32) | 432 (10.82) | 422 (10.57) | 0.7694 |
| other(current or previous) | 3581 (89.68) | 3562 (89.18) | 3572 (89.43) |  |
| Drinking Status (%) |  |  |  |  |
| never | 2381 (61.59) | 2330 (61.01) | 2344 (61.38) | 0.8709 |
| other(some or too much) | 1485 (38.41) | 1489 (38.99) | 1475 (38.62) |  |
| Diet Status |  |  |  |  |
| healthy | 624 (15.63) | 619 (15.50) | 599 (15.00) | 0.7119 |
| unhealthy | 3369 (84.37) | 3375 (84.50) | 3395 (85.00) |  |
| Physical Activity Level (%) |  |  |  |  |
| low | 1056 (26.45) | 1045 (26.16) | 1043 (26.11) | 0.936 |
| middle and high | 2937 (73.55) | 2949 (73.84) | 2951 (73.89) |  |
| Number of favorable lifestyle factors |  |  |  |  |
| 0 | 48 (1.20) | 45 (1.13) | 55 (1.38) | 0.5925 |
| 1 | 656 (16.43) | 629 (15.75) | 596 (14.92) |  |
| 2 | 1648 (41.27) | 1669 (41.79) | 1712 (42.86) |  |
| 3 | 1257 (31.48) | 1267 (31.72) | 1278 (32.00) |  |
| 4 | 350 (8.77) | 344 (8.61) | 313 (7.84) |  |
| 5 | 34 (0.85) | 40 (1.00) | 40 (1.00) |  |
| Lifestyle Category |  |  |  |  |
| unfavorable | 2352 (58.90) | 2343 (58.66) | 2363 (59.16) | 0.7406 |
| intermediate | 1257 (31.48) | 1267 (31.72) | 1278 (32.00) |  |
| favorable | 384 (9.62) | 384 (9.61) | 353 (8.84) |  |

Supplementary Table 4. Baseline characteristics of participants according to different lifestyle categories

| Characteristic | Unfavorable  (n=7058) | Intermediate  (n=3802) | Favorable  (n=1121) | P value |
| --- | --- | --- | --- | --- |
| Sex (%) |  |  |  |  |
| female | 2171 (30.76) | 1493 (39.27) | 530 (47.28) | <0.0001 |
| male | 4887 (69.24) | 2309 (60.73) | 591 (52.72) | |
| Baseline Age (median [IQR]) | 61.50[55.86,65.51] | 60.96[54.69,65.16] | 61.47[55.25,65.46] | <0.001 |
| Age Group |  |  |  |  |
| < 45 | 250 (3.54) | 185 (4.87) | 53 (4.73) | <0.001 |
| 45-60 | 2635 (37.33) | 1519 (39.95) | 414 (36.93) |  |
| > 60 | 4173 (59.12) | 2098 (55.18) | 654 (58.34) |  |
| TDI (median [IQR]) | -1.68 [-3.38, 1.43] | -2.06[-3.58,0.73] | -2.45 [-3.81,-0.04] | <0.0001 |
| Educational Level (%) | |  |  |  |
| A_O_CSE | 2561 (36.29) | 1385 (36.43) | 393 (35..06) | <0.0001 |
| NVQ_HND_HNC_professiol | 1084 (15.36) | 553 (14.54) | 143 (12.76) | |
| college | 1574 (22.30) | 1110 (29.20) | 411 (36.66) | |
| NA | 1839 (26.06) | 754 (19.83) | 174 (15.52) | |
| Income (%) | |  |  |  |
| low | 2037 (28.86) | 935 (24.59) | 236 (21.06) | <0.0001 |
| medium | 3129 (44.33) | 1755 (46.16) | 530 (47.28) | |
| high | 1049 (14.86) | 639 (16.81) | 197 (15.57) | |
| NA | 843 (11.94) | 473 (12.44) | 158 (14.09) |  |
| Employment status | |  |  |  |
| unemployment | 952 (13.49) | 337（8.86） | 78(6.96) | <0.0001 |
| employment | 6106 (86.51) | 3465 (91.14) | 1043 (93.04) | |
| Hypertension (%) |  |  |  |  |
| yes | 2200 (31.17) | 1457 (38.32) | 541 (48.26) | <0.0001 |
| no | 4858 (68.83) | 2345 (61.68) | 580 (51.74) |  |
| HbA1c (%) |  |  |  |  |
| < 7% | 4062 (60.01) | 2301 (62.68) | 692 (65.04) | <0.001 |
| ≥ 7% | 2707 (39.99) | 1370 (37.32) | 372 (34.96) |  |
| DM Duration (%) |  |  |  |  |
| < 3 years | 3817 (54.08) | 2035 (53.52) | 575 (51.29) | 0.2173 |
| ≥ 3 years | 3241 (45.92) | 1767 (46.48) | 546 (48.71) |  |
| BMI Type (%) |  |  |  |  |
| <25 | 184 (2.61) | 569 (14.97) | 659 (58.79) | <0.0001 |
| ≥25 | 6874 (97.39) | 3233 (85.03) | 462 (41.21) | |
| Smoking Status (%) | |  |  |  |
| never | 1362 (19.30) | 2749 (72.30) | 1026 (91.53) | <0.0001 |
| other(current or previous) | 5696 (80.70) | 1053 (27.70) | 95 (8.47) |  |
| Drinking Status (%) | |  |  |  |
| never | 1098 (15.56) | 148 (3.89) | 20 (1.78) | <0.0001 |
| other(some or too much) | 5960 (84.44) | 3654 (96.11) | 1101 (98.22) | |
| Diet Status | |  |  |  |
| healthy | 313 (4.43) | 828 (21.78) | 701 (62.53) | <0.0001 |
| unhealthy | 6745 (95.57) | 2974 (78.22) | 420 (37.47) | |
| Physical Activity Level (%) | | |  |  |
| low | 2938 (41.63) | 196 (5.16) | 10 (0.89) | <0.0001 |
| middle and high | 4120 (58.37) | 3606 (94.84) | 1111 (99.11) | |
| Genetic Risk Category | |  |  |  |
| low | 2352 (33.32) | 1257 (33.06) | 384 (34.26) | 0.7406 |
| moderate | 2343 (33.20) | 1267 (33.32) | 384 (34.26) | |
| high | 2363 (33.48) | 1278 (33.61) | 353 (31.49) | |

Supplementary Table 5A. Risk of DKD with Number of favorable lifestyle factors

| Number of favorable lifestyle factors | Events/Person-years | beta | HR | CI95 | P |
| --- | --- | --- | --- | --- | --- |
| 0 | 22/1922.915 | 0 | 1 | (ref) |  |
| 1 | 267/25423.68 | -0.1 | 0.9 | 0.58 - 1.39 | 0.642 |
| 2 | 611/69132.52 | -0.27 | 0.76 | 0.50 - 1.16 | 0.207 |
| 3 | 360/53386.92 | -0.56 | 0.57 | 0.37 - 0.88 | P<0.05 |
| 4 | 68/14671.95 | -0.97 | 0.38 | 0.23 - 0.61 | P<0.001 |
| 5 | 7/1652.562 | -1.02 | 0.36 | 0.15 - 0.84 | P<0.05 |
| Continuous variable* |  | -0.25 | 0.78 | 0.73-0.83 | P<0.001 |

Supplementary Table 5B. Risk of DKD with Number of favorable lifestyle factors

| Number of unfavorable lifestyle factors | Events/Person-years | beta | HR | CI95 | P |
| --- | --- | --- | --- | --- | --- |
| 0 | 7/1652.562 | 0 | 1 | (ref) |  |
| 1 | 68/14671.95 | 0.05 | 1.05 | 0.48-2.29 | 0.898 |
| 2 | 360/53386.92 | 0.46 | 1.59 | 0.75-2.36 | 0.225 |
| 3 | 611/69132.52 | 0.75 | 2.12 | 1.01-4.46 | P<0.05 |
| 4 | 267/25423.68 | 0.92 | 2.51 | 1.19-5.32 | P<0.05 |
| 5 | 7/1652.562 | 1.02 | 2.78 | 1.19-6.52 | P<0.05 |
| Continuous variable* |  | 0.25 | 1.29 | 1.21-1.37 | P<0.001 |

*The number of unfavorable lifestyle factors was used as a continuous variable for Cox regression analysis

Supplementary Table 6A. Risk of DKD according to each lifestyle factor, genetic risk and lifestyle categories within the male group

| Lifestyle factors | Model 1 | | Model 2 | | Model 3 | | Model 4 | |
| --- | --- | --- | --- | --- | --- | --- | --- | --- |
|  | HR (95%CI) | *P* value | HR (95%CI) | *P* value | HR (95%CI) | *P* value | HR (95%CI) | *P* value |
| BMI Type |  |  |  |  |  |  |  |  |
| ＜25 | 1 (ref) |  | 1 (ref) |  | 1 (ref) |  | 1 (ref) |  |
| ≥25 | 1.48 (1.16-1.89) | < 0.01 | 1.54 (1.20-1.97) | < 0.001 | 1.46 (1.14-1.87) | < 0.01 | 0.99 (0.27-1.64) | 0.062 |
| Smoking Status |  |  |  |  |  |  |  |  |
| never | 1 (ref) |  | 1 (ref) |  | 1 (ref) |  | 1 (ref) |  |
| other(current or previous) | 1.48 (1.29-1.71) | < 0.001 | 1.33 (1.16-1.53) | < 0.001 | 1.26 (1.09-1.46) | < 0.001 | 1.24 (1.08-1.44) | < 0.01 |
| Drinking Status (%) |  |  |  |  |  |  |  |  |
| never | 1 (ref) |  | 1 (ref) |  | 1 (ref) |  | 1 (ref) |  |
| other(some or too much) | 0.80 (0.64-0.98) | < 0.05 | 0.76 (0.61-0.94) | < 0.05 | 0.86 (0.69-1.07) | 0.176 | 0.86 (0.69-1.07) | 0.172 |
| Diet Status |  |  |  |  |  |  |  |  |
| healthy | 1 (ref) |  | 1 (ref) |  | 1 (ref) |  | 1 (ref) |  |
| unhealthy | 1.06 (0.88-1.27) | 0.543 | 1.13 (0.94-1.36) | 0.198 | 1.13 (0.94-1.36) | 0.206 | 1.11 (0.92-1.35) | 0.259 |
| Physical Activity Level |  |  |  |  |  |  |  |  |
| low | 1 (ref) |  | 1 (ref) |  | 1 (ref) |  | 1 (ref) |  |
| middle or high | 0.88 (0.77-1.02) | 0.093 | 0.85 (0.74-0.98) | < 0.05 | 0.88 (0.76-1.01) | 0.073 | 0.89 (0.77-1.02) | 0.102 |
| Genetic Risk Category |  |  |  |  |  |  |  |  |
| Low | 1 (ref) |  | 1 (ref) |  | 1 (ref) |  | 1 (ref) |  |
| Moderate | 1.15 (0.98-1.36) | 0.098 | 1.17 (0.99-1.37) | 0.061 | 1.16 (0.99-1.37) | 0.067 | 1.16 (0.99-1.37) | 0.069 |
| High | 1.23 (1.04-1.44) | < 0.05 | 1.25 (1.07-1.47) | < 0.01 | 1.28 (1.09-1.51) | < 0.01 | 1.31 (1.11-1.54) | < 0.01 |
| Lifestyle Category |  |  |  |  |  |  |  |  |
| Unfavorable | 1 (ref) |  | 1 (ref) |  | 1 (ref) |  | 1 (ref) |  |
| Intermediate | 0.71 (0.62-0.84) | < 0.001 | 0.74 (0.64-0.86) | < 0.001 | 0.77 (0.66-0.90) | < 0.001 | 0.79 (0.68-0.92) | < 0.01 |
| Favorable | 0.55 (0.41-0.73) | < 0.001 | 0.54 (0.40-0.73) | < 0.001 | 0.59 (0.44-0.79) | < 0.001 | 0.66 (0.49-0.89) | < 0.01 |
| The genetic risk-lifestyle categories |  |  |  |  |  |  |  |  |
| High-Unfavorable | 1 (ref) |  | 1 (ref) |  | 1 (ref) |  | 1 (ref) |  |
| High-Intermediate | 0.53 (0.40-0.70) | < 0.001 | 0.54 (0.41-0.71) | < 0.001 | 0.57 (0.43-0.75) | < 0.001 | 0.58 (0.44-0.77) | < 0.001 |
| High-Favorable | 0.63 (0.40-0.99) | < 0.05 | 0.63-0.40-0.99) | < 0.05 | 0.69 (0.44-1.09) | 0.113 | 0.82 (0.52-1.29) | 0.392 |
| Moderate-Unfavorable | 0.87 (0.73-1.04) | 0.133 | 0.85 (0.71-1.02) | 0.081 | 0.83 (0.69-0.99) | < 0.05 | 0.82 (0.68-0.99) | < 0.05 |
| Moderate -Intermediate | 0.69 (0.54-0.88) | < 0.01 | 0.71 (0.56-0.91) | < 0.01 | 0.72 (0.56-0.92) | < 0.01 | 0.72 (0.56-0.93) | < 0.05 |
| Moderate -Favorable | 0.44 (0.26-0.75) | < 0.01 | 0.44 (0.26-0.74) | < 0.01 | 0.48 (0.28-0.80) | < 0.01 | 0.49 (0.29-0.85) | < 0.05 |
| Low-Unfavorable | 0.73 (0.60-0.89) | < 0.01 | 0.72 (0.60-0.87) | < 0.001 | 0.70 (0.58-0.85) | < 0.001 | 0.69 (0.57-0.84) | < 0.001 |
| Low-Intermediate | 0.65 (0.51-0.84) | < 0.001 | 0.65 (0.51-0.84) | < 0.001 | 0.67 (0.52-0.86) | < 0.01 | 0.67 (0.51-0.86) | < 0.01 |
| Low-Favorable | 0.36 (0.21-0.63) | < 0.001 | 0.34 (0.20-0.60) | < 0.001 | 0.36 (0.21-0.63) | < 0.001 | 0.40 (0.23-0.70) | < 0.01 |

Model 1：No adjusted

Model 2：Adjusted for age and sex

Model 3：Adjusted for covariates in model2 and TDI, Income, Educational Level, Employment status.

Model 4：Adjusted for covariates in model3 and comorbidity with hypertension, DM duration, HbA1c.

# Encode the ordered categorical variables 1, 2, and 3 as continuous variables and include them in the regression model

Supplementary Table 6B. Risk of DKD according to each lifestyle factor, genetic risk and lifestyle categories within the female group

| Lifestyle factors | Model 1 | | Model 2 | | Model 3 | | Model 4 | |
| --- | --- | --- | --- | --- | --- | --- | --- | --- |
|  | HR (95%CI) | *P* value | HR (95%CI) | *P* value | HR (95%CI) | *P* value | HR (95%CI) | *P* value |
| BMI Type |  |  |  |  |  |  |  |  |
| < 25 | 1 (ref) |  | 1 (ref) |  | 1 (ref) |  | 1 (ref) |  |
| ≥ 25 | 2.07 (1.46-2.94) | < 0.001 | 1.99 (1.40-2.83) | < 0.001 | 1.85 (1.30-2.63) | < 0.001 | 1.66 (1.16-2.38) | < 0.01 |
| Smoking Status |  |  |  |  |  |  |  |  |
| never | 1 (ref) |  | 1 (ref) |  | 1 (ref) |  | 1 (ref) |  |
| other(current or previous) | 1.30 (1.07-1.57) | < 0.01 | 1.25 (1.03-1.52) | < 0.05 | 1.17 (0.96-1.42) | 0.122 | 1.13 (0.93-1.39) | 0.22 |
| Drinking Status (%) |  |  |  |  |  |  |  |  |
| never | 1 (ref) |  | 1 (ref) |  | 1 (ref) |  | 1 (ref) |  |
| other(some or too much) | 0.59 (0.47-0.74) | < 0.001 | 0.65 (0.51-0.82) | < 0.001 | 0.70 (0.55-0.86) | < 0.01 | 0.73 (0.57-0.93) | < 0.01 |
| Diet Status |  |  |  |  |  |  |  |  |
| healthy | 1 (ref) |  | 1 (ref) |  | 1 (ref) |  | 1 (ref) |  |
| unhealthy | 1.02 (0.79-1.31) | 0.921 | 1.14 (0.89-1.47) | 0.306 | 1.15 (0.89-1.49) | 0.274 | 1.15 (0.88-1.49) | 0.303 |
| Physical Activity Level |  |  |  |  |  |  |  |  |
| low | 1 (ref) |  | 1 (ref) |  | 1 (ref) |  | 1 (ref) |  |
| middle or high | 0.83 (0.67-1.03) | 0.09 | 0.80 (0.64-0.98) | < 0.005 | 0.82 (0.66-1.01) | 0.063 | 0.84 (0.68-1.05) | 0.123 |
| Genetic Risk Category |  |  |  |  |  |  |  |  |
| Low | 1 (ref) |  | 1 (ref) |  | 1 (ref) |  | 1 (ref) |  |
| Moderate | 1.24 (0.96-1.60) | 0.098 | 1.30 (1.00-1.66) | < 0.05 | 1.30 (1.01-1.68) | < 0.05 | 1.33 (1.03-1.73) | < 0.05 |
| High | 1.47 (1.16-1.88) | < 0.01 | 1.57 (1.22-2.00) | < 0.001 | 1.57 (1.23-2.01) | < 0.001 | 1.63 (1.27-2.10) | < 0.001 |
| Lifestyle Category |  |  |  |  |  |  |  |  |
| Unfavorable | 1 (ref) |  | 1 (ref) |  | 1 (ref) |  | 1 (ref) |  |
| Intermediate | 0.73 (0.59-0.90) | < 0.01 | 0.73 (0.59-0.91) | < 0.01 | 0.77 (0.62-0.95) | < 0.01 | 0.81 (0.65-1.01) | 0.062 |
| Favorable | 0.40 (0.27-0.60) | < 0.001 | 0.39 (0.26-0.58) | < 0.001 | 0.42 (0.28-0.63) | < 0.001 | 0.46 (0.31-0.69) | < 0.001 |
| The genetic risk-lifestyle categories |  |  |  |  |  |  |  |  |
| High-Unfavorable | 1 (ref) |  | 1 (ref) |  | 1 (ref) |  | 1 (ref) |  |
| High-Intermediate | 0.80 (0.58-1.11) | 0.185 | 0.81 (0.58-1.12) | 0.203 | 0.84 (0.60-1.16) | 0.288 | 0.88 (0.63-1.23) | 0.460 |
| High-Favorable | 0.39 (0.20-0.75) | < 0.01 | 0.40 (0.21-0.78) | < 0.01 | 0.44 (0.23-0.85) | < 0.05 | 0.49 (0.25-0.94) | < 0.05 |
| Moderate -Unfavorable | 0.88 (0.66-1.18) | 0.408 | 0.88 (0.65-1.17) | 0.369 | 0.88 (0.66-1.18) | 0.401 | 0.88 (0.65-1.18) | 0.388 |
| Moderate -Intermediate | 0.58 (0.40-0.84) | < 0.01 | 0.59 (0.41-0.85) | < 0.01 | 0.62 (0.43-0.89) | < 0.05 | 0.63 (0.43-0.92) | < 0.05 |
| Moderate -Favorable | 0.41 (0.22-0.76) | < 0.01 | 0.38 (0.20-0.71) | < 0.01 | 0.40 (0.22-0.76) | < 0.01 | 0.43 (0.22-0.83) | < 0.05 |
| Low-Unfavorable | 0.71 (0.52-0.97) | < 0.05 | 0.69 (0.50-0.94) | < 0.05 | 0.67 (0.49-0.92) | < 0.05 | 0.65 (0.47-0.89) | < 0.01 |
| Low-Intermediate | 0.50 (0.33-0.74) | < 0.001 | 0.47 (0.31-0.70) | < 0.001 | 0.50 (0.33-0.74) | < 0.001 | 0.51 (0.34-0.77) | < 0.01 |
| Low-Favorable | 0.24 (0.11-0.55) | < 0.001 | 0.22 (0.10-0.50) | < 0.001 | 0.24 (0.11-0.55) | < 0.001 | 0.26 (0.11-0.59) | < 0.01 |

Model 1：No adjusted

Model 2：Adjusted for age and sex

Model 3：Adjusted for covariates in model2 and TDI, Income, Educational Level, Employment status.

Model 4：Adjusted for covariates in model3 and comorbidity with hypertension, DM duration, HbA1c.

# Encode the ordered categorical variables 1, 2, and 3 as continuous variables and include them in the regression model

Supplementary Table 7. The sensitivity analysis with respect to BMI

| Lifestyle factors | Model 1 | | Model 2 | | Model 3 | | Model 4 | |
| --- | --- | --- | --- | --- | --- | --- | --- | --- |
|  | HR (95%CI) | P value | HR (95%CI) | P value | HR (95%CI) | P value | HR (95%CI) | P value |
| BMI Type |  |  |  |  |  |  |  |  |
| < 30 | 1 (ref) |  | 1 (ref) |  | 1 (ref) |  | 1 (ref) |  |
| ≥ 30 | 1.40 (1.25-1.56) | < 0.001 | 1.52 (1.36-1.70) | < 0.001 | 1.44 (1.29-1.61) | < 0.001 | 1.31 (1.17-1.47) | < 0.001 |
| Lifestyle Category |  |  |  |  |  |  |  |  |
| Unfavorable | 1 (ref) |  | 1 (ref) |  | 1 (ref) |  | 1 (ref) |  |
| Intermediate | 0.79 (0.70-0.89) | < 0.001 | 0.78 (0.70-0.88) | < 0.001 | 0.82 (0.72-0.92) | < 0.001 | 0.84 (0.75-0.95) | < 0.01 |
| Favorable | 0.52 (0.45-0.62) | < 0.001 | 0.51 (0.44-0.60) | < 0.001 | 0.56 (0.47-0.66) | < 0.001 | 0.61 (0.52-0.72) | < 0.001 |
| High genetic risk |  |  |  |  |  |  |  |  |
| Unfavorable lifestyle | 1 (ref) |  | 1 (ref) |  | 1 (ref) |  | 1 (ref) |  |
| Immediate lifestyle | 0.69 (0.57-0.84) | < 0.001 | 0.69 (0.56-0.83) | < 0.001 | 0.72 (0.59-0.88) | < 0.01 | 0.75 (0.61-0.91) | < 0.01 |
| Favorable lifestyle | 0.44 (0.33-0.57) | < 0.001 | 0.43 (0.33-0.57) | < 0.001 | 0.48 (0.36-0.63) | < 0.001 | 0.53 (0.40-0.70) | < 0.001 |
| *P* value for trend |  | < 0.001 |  | < 0.001 |  | < 0.001 |  | < 0.001 |
| Moderate Genetic Risk |  |  |  |  |  |  |  |  |
| Unfavorable lifestyle | 1 (ref) |  | 1 (ref) |  | 1 (ref) |  | 1 (ref) |  |
| Immediate lifestyle | 0.88 (0.72-1.08) | 0.215 | 0.89 (0.73-1.09) | 0.247 | 0.92 (0.75-1.12) | 0.408 | 0.94 (0.77-1.16) | 0.559 |
| Favorable lifestyle | 0.54 (0.41-0.71) | < 0.001 | 0.54 (0.41-0.71) | < 0.001 | 0.57 (0.43-0.75) | < 0.001 | 0.63 (0.47-0.83) | < 0.01 |
| *P* value for trend |  | < 0.001 |  | < 0.001 |  | < 0.001 |  | < 0.01 |
| Low Genetic Risk |  |  |  |  |  |  |  |  |
| Unfavorable lifestyle | 1 (ref) |  | 1 (ref) |  | 1 (ref) |  | 1 (ref) |  |
| Immediate lifestyle | 0.83 (0.67-1.04) | 0.106 | 0.80 (0.64-0.99) | < 0.05 | 0.83 (0.67-1.04) | 0.111 | 0.86 (0.68-1.08) | 0.205 |
| Favorable lifestyle | 0.63 (0.48-0.84) | < 0.01 | 0.60 (0.45-0.80) | < 0.001 | 0.66 (0.49-0.88) | < 0.01 | 0.71 (0.53-0.96) | < 0.05 |
| *P* value for trend |  | < 0.01 |  | < 0.001 |  | < 0.01 |  | < 0.05 |
| The genetic risk-lifestyle categories |  |  |  |  |  |  |  |  |
| High-Unfavorable | 1 (ref) |  | 1 (ref) |  | 1 (ref) |  | 1 (ref) |  |
| High-Intermediate | 0.69 (0.57-0.84) | < 0.001 | 0.69 (0.57-0.84) | < 0.001 | 0.71 (0.59-0.87) | < 0.001 | 0.75 (0.61-0.91) | < 0.01 |
| High-Favorable | 0.44 (0.33-0.58) | < 0.001 | 0.44 (0.33-0.57) | < 0.001 | 0.48 (0.36-0.63) | < 0.001 | 0.53 (0.40-0.71) | < 0.001 |
| Moderate-Unfavorable | 0.81 (0.68-0.97) | < 0.05 | 0.80 (0.67-0.95) | < 0.05 | 0.78 (0.66-0.94) | < 0.01 | 0.78 (0.65-0.94) | < 0.01 |
| Moderate -Intermediate | 0.72 (0.59-0.87) | < 0.001 | 0.71 (0.58-0.86) | < 0.001 | 0.73 (0.60-0.88) | < 0.01 | 0.74 (0.61-0.90) | < 0.01 |
| Moderate -Favorable | 0.44 (0.33-0.57) | < 0.001 | 0.43 (0.32-0.56) | < 0.001 | 0.45 (0.35-0.59) | < 0.001 | 0.49 (0.37-0.65) | < 0.001 |
| Low-Unfavorable | 0.69 (0.57-0.83) | < 0.001 | 0.68 (0.56-0.82) | < 0.001 | 0.66 (0.55-0.80) | < 0.001 | 0.65 (0.54-0.79) | < 0.001 |
| Low-Intermediate | 0.58 (0.47-0.71) | < 0.001 | 0.55 (0.44-0.67) | < 0.001 | 0.58 (0.45-0.69) | < 0.001 | 0.57 (0.46-0.70) | < 0.001 |
| Low-Favorable | 0.44 (0.33-0.58) | < 0.001 | 0.41 (0.31-0.54) | < 0.001 | 0.44 (0.33-0.58) | < 0.001 | 0.46 (0.35-0.62) | < 0.001 |

Model 1：No adjusted

Model 2：Adjusted for age and sex

Model 3：Adjusted for covariates in model2 and TDI, Income, Educational Level, Employment status.

Model 4：Adjusted for covariates in model3 and comorbidity with hypertension, DM duration, HbA1c.

Supplementary Table 8. The sensitivity analysis with respect to UACR

| Lifestyle factors | Model 1 | | | | Model 2 | | | Model 3 | | | | Model 4 | | |
| --- | --- | --- | --- | --- | --- | --- | --- | --- | --- | --- | --- | --- | --- | --- |
|  | HR (95%CI) | | | *P* value | HR (95%CI) | | *P* value | HR (95%CI) | | *P* value | | HR (95%CI) | | *P* value |
| BMI Type |  | | |  |  | |  |  | |  | |  | |  |
| < 25 | 1 (ref) | | |  | 1 (ref) | |  | 1 (ref) | |  | | 1 (ref) | |  |
| ≥ 25 | 1.75 (1.42-2.16) | | | < 0.001 | 1.73 (1.40-2.13) | | < 0.001 | 1.64 (1.33-2.02) | | < 0.001 | | 1.45 (1.17-1.80) | | < 0.001 |
| Smoking Status |  | | |  |  | |  |  | |  | |  | |  |
| never | 1 (ref) | | |  | 1 (ref) | |  | 1 (ref) | |  | | 1 (ref) | |  |
| other(current or previous) | 1.43 (1.28-1.61) | | | < 0.001 | 1.27 (1.13-1.43) | | < 0.001 | 1.20 (1.07-1.35) | | < 0.01 | | 1.18 (1.05-1.33) | | < 0.01 |
| Drinking Status (%) |  | | |  |  | |  |  | |  | |  | |  |
| never | 1 (ref) | | |  | 1 (ref) | |  | 1 (ref) | |  | | 1 (ref) | |  |
| other(some or too much) | 0.74 (0.63-0.87) | | | < 0.001 | 0.72 (0.61-0.85) | | < 0.001 | 0.80 (0.68-0.94) | | < 0.01 | | 0.81 (0.69-0.96) | | < 0.05 |
| Diet Status |  | | |  |  | |  |  | |  | |  | |  |
| healthy | 1 (ref) | | |  | 1 (ref) | |  | 1 (ref) | |  | | 1 (ref) | |  |
| unhealthy | 1.08 (0.93-1.26) | | | 0.324 | 1.15 (0.99-1.35) | | 0.073 | 1.15 (0.98-1.34) | | 0.078 | | 1.14 (0.97-1.34) | | 0.102 |
| Physical Activity Level |  | | |  |  | |  |  | |  | |  | |  |
| low | 1 (ref) | | |  | 1 (ref) | |  | 1 (ref) | |  | | 1 (ref) | |  |
| middle or high | 0.87 (0.77-0.99) | | | < 0.05 | 0.84 (0.74-0.95) | | < 0.005 | 0.85 (0.76-0.98) | | < 0.05 | | 0.86 (0.76-0.98) | | < 0.05 |
| Genetic Risk Category |  | | |  |  | |  |  | |  | |  | |  |
| Low | 1 (ref) | | |  | 1 (ref) | |  | 1 (ref) | |  | | 1 (ref) | |  |
| Moderate | 1.16 (1.01-1.34) | | | < 0.05 | 1.19 (1.04-1.37) | | < 0.05 | 1.20 (1.04-1.38) | | < 0.05 | | 1.21 (1.05-1.40) | | < 0.01 |
| High | 1.29 (1.12-1.48) | | | < 0.001 | 1.34 (1.16-1.54) | | < 0.001 | 1.37 (1.19-1.57) | | < 0.001 | | 1.41 (1.22-1.62) | | < 0.001 |
| Lifestyle Category |  | | |  |  | |  |  | |  | |  | |  |
| Unfavorable | 1 (ref) | | |  | 1 (ref) | |  | 1 (ref) | |  | | 1 (ref) | |  |
| Intermediate | 0.70 (0.62-0.80) | | | < 0.001 | 0.73 (0.65-0.83) | | < 0.001 | 0.78 (0.68-0.87) | | < 0.001 | | 0.79 (0.69-0.90) | | < 0.001 |
| Favorable | 0.47 (0.37-0.60) | | | < 0.001 | 0.50 (0.38-0.61) | | < 0.001 | 0.52 (0.41-0.66) | | < 0.001 | | 0.59 (0.44-0.73) | | < 0.001 |
| High genetic risk | |  |  | | |  |  | |  | |  | |  |  |
| Unfavorable lifestyle | | 1 (ref) |  | | | 1 (ref) |  | | 1 (ref) | |  | | 1 (ref) |  |
| Immediate lifestyle | | 0.58 (0.47-0.73) | < 0.001 | | | 0.60 (0.48-0.75) | < 0.001 | | 0.64 (0.51-0.79) | | < 0.001 | | 0.66 (0.53-0.82) | < 0.001 |
| Favorable lifestyle | | 0.50 (0.34-0.73) | < 0.001 | | | 0.52 (0.35-0.77) | < 0.001 | | 0.58 (0.39-0.86) | | < 0.01 | | 0.66 (0.44-0.97) | < 0.05 |
| *P* value for trend | |  | < 0.001 | | |  | < 0.001 | |  | | < 0.001 | |  | < 0.001 |
| Moderate Genetic Risk | |  |  | | |  |  | |  | |  | |  |  |
| Unfavorable lifestyle | | 1 (ref) |  | | | 1 (ref) |  | | 1 (ref) | |  | | 1 (ref) |  |
| Immediate lifestyle | | 0.74 (0.59-0.91) | < 0.01 | | | 0.78 (0.63-0.97) | < 0.05 | | 0.79 (0.64-0.99) | | < 0.05 | | 0.81 (0.65-1.01) | 0.062 |
| Favorable lifestyle | | 0.51 (0.34-0.76) | < 0.001 | | | 0.53 (0.35-0.79) | < 0.001 | | 0.56 (0.38-0.85) | | < 0.01 | | 0.59 (0.39-0.91) | < 0.05 |
| *P* value for trend | |  | < 0.001 | | |  | < 0.001 | |  | | < 0.01 | |  | < 0.01 |
| Low Genetic Risk | |  |  | | |  |  | |  | |  | |  |  |
| Unfavorable lifestyle | | 1 (ref) |  | | | 1 (ref) |  | | 1 (ref) | |  | | 1 (ref) |  |
| Immediate lifestyle | | 0.83 (0.66-1.05) | 0.115 | | | 0.85 (0.68-1.07) | 0.165 | | 0.90 (0.71-1.13) | | 0.350 | | 0.91 (0.72-1.15) | 0.434 |
| Favorable lifestyle | | 0.39 (0.24-0.65) | < 0.001 | | | 0.39 (0.24-0.64) | < 0.001 | | 0.41 (0.25-0.68) | | < 0.001 | | 0.46 (0.28-0.76) | < 0.01 |
| *P* value for trend | |  | < 0.01 | | |  | < 0.001 | |  | | < 0.01 | |  | < 0.01 |
| The genetic risk-lifestyle categories |  | | |  |  | |  |  | |  | |  | |  |
| High-Unfavorable | 1 (ref) | | |  | 1 (ref) | |  | 1 (ref) | |  | | 1 (ref) | |  |
| High-Intermediate | 0.59 (0.47-0.73) | | | < 0.001 | 0.61 (0.49-0.76) | | < 0.001 | 0.62 (0.50-0.77) | | < 0.001 | | 0.65 (0.52-0.81) | | < 0.001 |
| High-Favorable | 0.50 (0.34-0.74) | | | < 0.001 | 0.53 (0.36-0.78) | | < 0.01 | 0.55 (0.37-0.81) | | < 0.01 | | 0.64 (0.44-0.95) | | < 0.05 |
| Moderate -Unfavorable | 0.85 (0.73-0.99) | | | < 0.05 | 0.84 (0.71-0.98) | | < 0.05 | 0.83 (0.71-0.97) | | < 0.05 | | 0.82 (0.70-0.97) | | < 0.05 |
| Moderate -Intermediate | 0.63 (0.51-0.77) | | | < 0.001 | 0.65 (0.53-0.81) | | < 0.001 | 0.67 (0.54-0.83) | | < 0.001 | | 0.68 (0.55-0.84) | | < 0.001 |
| Moderate -Favorable | 0.43 (0.29-0.44) | | | < 0.001 | 0.44 (0.29-0.65) | | < 0.001 | 0.45 (0.30-0.68) | | < 0.001 | | 0.48 (0.32-0.73) | | < 0.001 |
| Low-Unfavorable | 0.71 (0.60-0.85) | | | < 0.001 | 0.70 (0.59-0.83) | | < 0.001 | 0.69 (0.58-0.82) | | < 0.001 | | 0.67 (0.57-0.80) | | < 0.001 |
| Low-Intermediate | 0.60 (0.48-0.74) | | | < 0.001 | 0.59 (0.47-0.74) | | < 0.001 | 0.61 (0.49-0.76) | | < 0.001 | | 0.61 (0.48-0.76) | | < 0.001 |
| Low-Favorable | 0.28 (0.17-0.49) | | | < 0.001 | 0.27 (0.17-0.44) | | < 0.001 | 0.28 (0.17-0.45) | | < 0.001 | | 0.30 (0.19-0.50) | | < 0.001 |

Model 1：No adjusted

Model 2：Adjusted for age and sex

Model 3：Adjusted for covariates in model2 and TDI, Income, Educational Level, Employment status.

Model 4：Adjusted for covariates in model3 and comorbidity with hypertension, DM duration, HbA1c.
